# The paradigm change from reactive medical services to 3PM in ischemic stroke: a holistic approach utilising tear fluid multi-omics, mitochondria as a vital biosensor and AI-based multi-professional data interpretation

**DOI:** 10.1007/s13167-024-00356-6

**Published:** 2024-02-27

**Authors:** Olga Golubnitschaja, Jiri Polivka, Pavel Potuznik, Martin Pesta, Ivana Stetkarova, Alena Mazurakova, Lenka Lackova, Peter Kubatka, Martina Kropp, Gabriele Thumann, Carl Erb, Holger Fröhlich, Wei Wang, Babak Baban, Marko Kapalla, Niva Shapira, Kneginja Richter, Alexander Karabatsiakis, Ivica Smokovski, Leonard Christopher Schmeel, Eleni Gkika, Friedemann Paul, Paolo Parini, Jiri Polivka

**Affiliations:** 1 Predictive, Preventive and Personalised (3P) Medicine, Department of Radiation Oncology, University Hospital Bonn, Rheinische Friedrich-Wilhelms-Universität Bonn, 53127 Bonn, Germany; 2 Department of Histology and Embryology, Faculty of Medicine in Plzen, Charles University, Prague, Czech Republic; 3 Biomedical Centre, Faculty of Medicine in Plzen, Charles University, Prague, Czech Republic; 4 Department of Neurology, University Hospital Plzen and Faculty of Medicine in Plzen, Charles University, Prague, Czech Republic; 5 Department of Biology, Faculty of Medicine in Plzen, Charles University, Prague, Czech Republic; 6 Department of Neurology, University Hospital Kralovske Vinohrady, Third Faculty of Medicine, Charles University, Prague, Czech Republic; 7 Department of Anatomy, Jessenius Faculty of Medicine, Comenius University in Bratislava, Martin, Slovakia; 8 Department of Histology and Embryology, Jessenius Faculty of Medicine, Comenius University in Bratislava, Martin, Slovakia; 9 Experimental Ophthalmology, University of Geneva, 1205 Geneva, Switzerland; 10 Ophthalmology Department, University Hospitals of Geneva, 1205 Geneva, Switzerland; 11 Private Institute of Applied Ophthalmology, Berlin, Germany; 12 Artificial Intelligence & Data Science Group, Fraunhofer SCAI, Sankt Augustin, Germany; 13 Bonn-Aachen International Center for IT (B-It), University of Bonn, 53115 Bonn, Germany; 14 Edith Cowan University, Perth, Australia; 15 Beijing Municipal Key Laboratory of Clinical Epidemiology, Capital Medical University, Beijing, China; 16 The Dental College of Georgia, Departments of Neurology and Surgery, The Medical College of Georgia, Augusta University, Augusta, USA; 17 Negentropic Systems, Ružomberok, Slovakia; 18 PPPM Centre, s.r.o., Ruzomberok, Slovakia; 19 Department of Nutrition, School of Health Sciences, Ashkelon Academic College, Ashkelon, Israel; 20 CuraMed Tagesklinik Nürnberg GmbH, Nuremberg, Germany; 21 Technische Hochschule Nürnberg GSO, Nuremberg, Germany; 22 University Clinic for Psychiatry and Psychotherapy, Paracelsus Medical University, Nuremberg, Germany; 23 Department of Psychology, Clinical Psychology II, University of Innsbruck, Innsbruck, Austria; 24 University Clinic of Endocrinology, Diabetes and Metabolic Disorders Skopje, University Goce Delcev, Faculty of Medical Sciences, Stip, North Macedonia; 25 Department of Radiation Oncology, University Hospital Bonn, Rheinische Friedrich-Wilhelms-Universität Bonn, 53127 Bonn, Germany; 26 Charité University Medicine Berlin, Berlin, Germany; 27 Cardio Metabolic Unit, Department of Medicine Huddinge, and Department of Laboratory Medicine, Karolinska Institutet, and Medicine Unit of Endocrinology, Theme Inflammation and Ageing, Karolinska University Hospital, Stockholm, Sweden

**Keywords:** Predictive preventive personalised medicine (PPPM / 3PM), Ischemic stroke, Sudden cardiac arrest/death, Suboptimal health, Health-to-disease transition, Primary and secondary care, Patient-friendly non-invasive approach, Tear fluid analysis, Viromics and metabolomics, Mitochondrial health, Mitophagy, Inflammation, Cytokine storm (COVID-19), Diabetes mellitus, Diabetic retinopathy, Flammer syndrome, Health risk assessment, Sleep medicine, Behavioural patterns, Individualised patient profile, Artificial intelligence, Population screening, Healthcare economy, Health policy, Expert recommendations

## Abstract

Worldwide stroke is the second leading cause of death and the third leading cause of death and disability combined. The estimated global economic burden by stroke is over US$891 billion per year. Within three decades (1990–2019), the incidence increased by 70%, deaths by 43%, prevalence by 102%, and DALYs by 143%. Of over 100 million people affected by stroke, about 76% are ischemic stroke (IS) patients recorded worldwide. Contextually, ischemic stroke moves into particular focus of multi-professional groups including researchers, healthcare industry, economists, and policy-makers. Risk factors of ischemic stroke demonstrate sufficient space for cost-effective prevention interventions in primary (suboptimal health) and secondary (clinically manifested collateral disorders contributing to stroke risks) care. These risks are interrelated. For example, sedentary lifestyle and toxic environment both cause mitochondrial stress, systemic low-grade inflammation and accelerated ageing; inflammageing is a low-grade inflammation associated with accelerated ageing and poor stroke outcomes. Stress overload, decreased mitochondrial bioenergetics and hypomagnesaemia are associated with systemic vasospasm and ischemic lesions in heart and brain of all age groups including teenagers. Imbalanced dietary patterns poor in folate but rich in red and processed meat, refined grains, and sugary beverages are associated with hyperhomocysteinaemia, systemic inflammation, small vessel disease, and increased IS risks. Ongoing 3PM research towards vulnerable groups in the population promoted by the European Association for Predictive, Preventive and Personalised Medicine (EPMA) demonstrates promising results for the holistic patient-friendly non-invasive approach utilising tear fluid-based health risk assessment, mitochondria as a vital biosensor and AI-based multi-professional data interpretation as reported here by the EPMA expert group. Collected data demonstrate that IS-relevant risks and corresponding molecular pathways are interrelated. For examples, there is an evident overlap between molecular patterns involved in IS and diabetic retinopathy as an early indicator of IS risk in diabetic patients. Just to exemplify some of them such as the 5-aminolevulinic acid/pathway, which are also characteristic for an altered mitophagy patterns, insomnia, stress regulation and modulation of microbiota-gut-brain crosstalk. Further, ceramides are considered mediators of oxidative stress and inflammation in cardiometabolic disease, negatively affecting mitochondrial respiratory chain function and fission/fusion activity, altered sleep–wake behaviour, vascular stiffness and remodelling. Xanthine/pathway regulation is involved in mitochondrial homeostasis and stress-driven anxiety-like behaviour as well as molecular mechanisms of arterial stiffness. In order to assess individual health risks, an application of machine learning (AI tool) is essential for an accurate data interpretation performed by the multiparametric analysis. Aspects presented in the paper include the needs of young populations and elderly, personalised risk assessment in primary and secondary care, cost-efficacy, application of innovative technologies and screening programmes, advanced education measures for professionals and general population—all are essential pillars for the paradigm change from reactive medical services to 3PM in the overall IS management promoted by the EPMA.

## Ischemic stroke: alarming statistics and well-acknowledged risks

According to the most recent Global Burden of Disease (GBD) estimates, worldwide stroke is the second leading cause of death and the third leading cause of death and disability (see disability-adjusted life-years lost, DALYs statistics presented below) combined [1]. The estimated global economic burden by stroke is over US$891 billion per year comprising 1.12% of the global GDP [2]. Within three decades (years 1990–2019) considering the absolute number of all stroke cases, the burden increased as follows: incidence by 70%, deaths by 43%, prevalence by 102%, and DALYs by 143%. The age-standardised difference between countries is remarkable taking into consideration the six-fold incidence, 15-fold mortality, four-fold prevalence, and 20-fold DALYs rates, which are particularly high in lower- and middle-income countries situated in Eastern Europe, Asia, and Sub-Saharan Africa [1].

**Fig. 1:**
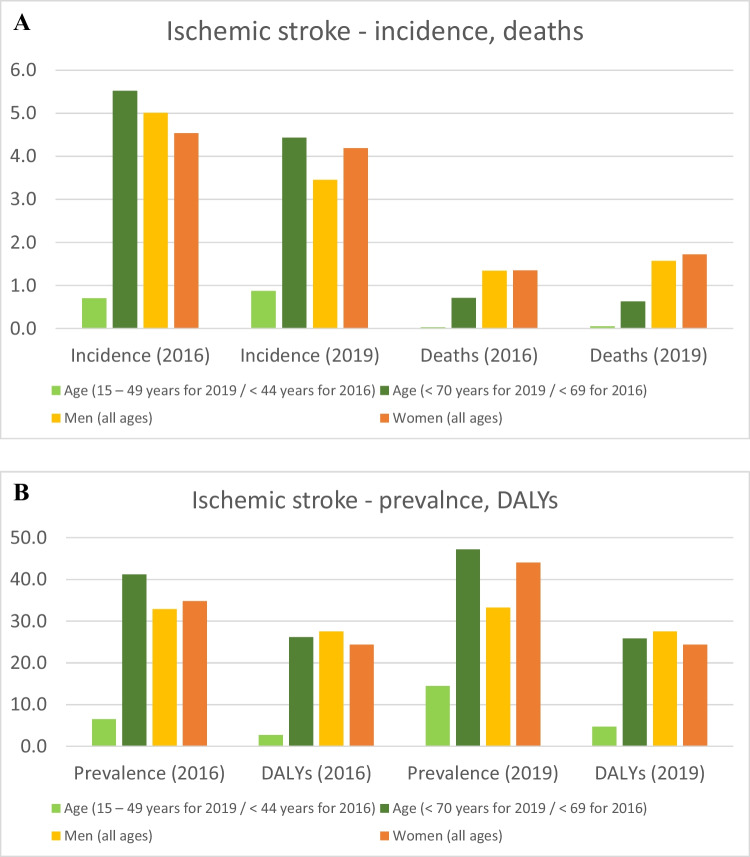
**A** Numbers (million) of ischemic stroke incidence and deaths amongst different age and sex categories in 2016 and 2019. **B** Numbers (million) of ischemic stroke prevalence and DALYs amongst different age and sex categories in 2016 and 2019

Of 101.5 million (2019) people affected by stroke, about 76% are ischemic stroke (IS) patients recorded worldwide. Table 1 and Fig. 1A and B both compare statistics originated from 2016 and 2019 (GBD) [1, 3–5]. From altogether, 13.68 million new strokes occurred in 2016, and 9.56 million (69.9%) accounted for IS with 51.9 million disability-adjusted life-years (DALYs) [3, 4]. Three years later (2019), IS-related deaths increased by 22.3% against 18.5% from all strokes, and DALYs increased by 23% for all strokes [1, 5]. Noteworthy, the IS prevalence increased for both sexes but more remarkably for females. Although all age groups demonstrate an increase in the IS prevalence, specifically the young (< 49 y.o.) stroke prevalence has doubled within 3 years followed by concomitant DALYs increase due to IS in this age group. Contextually, ischemic stroke moves into particular focus of multi-professional groups including researchers, healthcare industry, economists, and policy-makers. All aspects are of great importance, namely, the needs of young populations and elderly, personalised risk assessment in primary and secondary care, cost-efficacy, innovative screening programmes, and advanced education measures for professionals and general population, amongst others—altogether resulting in the paradigm change from reactive medical services to a predictive approach, targeted prevention, and treatments tailored to the person (predictive, preventive, and personalised medicine, shortly PPPM/3PM).

**Table 1:** Incidence, prevalence, deaths, and DALYs for stroke (all types) and ischemic stroke compared for the year 2019 versus 2016

	2016		2019	
	All stroke	Ischemic stroke (all ages, both sexes)	All stroke	Ischemic stroke (all ages, both sexes)
Incidence	13 676 761	9 556 444	12 224 551	7 630 803
Prevalence	80 065 453	67 595 368	101 474 558	77 192 498
Deaths	5 528 232	2 690 171	6 552 724	3 293 397
DALYs	116 445 136	51 897 437	143 232 184	63 478 271

## Multi-factorial risks of IS with sufficient space for preventive interventions

As presented by GBD 2016 Stroke Collaborators, 87.9% of ischemic stroke DALYs were associated with potentially preventable risks such as smoking, inappropriate dietary habits and behavioural patterns, physical inactivity, and toxic environmental, amongst others [1, 6]. Therefore, appropriate educational measures and improved lifestyle patterns in the population are considered a potentially effective strategy to reduce ischemic stroke mortality and morbidity [7]. However, health risk assessment should evaluate comprehensive individualised profiles including both modifiable and non-modifiable risk factors for cost-effective targeted prevention in primary (suboptimal health conditions with reversible damage to health) and secondary (clinically manifested disorders) care in the framework of 3PM.

According to the biopsychosocial model of health and disease, a multifactorial concept for the risk evaluation towards prevention and prediction have to be taken into consideration. For example, considering lifestyle factors, an imbalanced diet and inappropriate nutritional patterns are frequently observed in patients with clinically manifested IS compared to healthy controls and generally accepted Mediterranean diet [8–12]. Malnutrition, underweight, overweight, and obesity represent highly relevant risks of IS [10, 11, 13, 14]. The rise in adolescent obesity as well as underweight may significantly increase future young stroke prevalence [15–17]. Further, not the BMI deviation itself but rather individual metabolic consequences contribute to the stroke predisposition [13]. To this end, the “obesity paradox” has been described suggesting a lower risk of mortality and favourable outcomes in obese or overweight patients compared to the normal BMI range [16–21].

Physical inactivity/sedentary lifestyle is a strong contributor to IS [22, 23] as a multi-faceted risk factor including mitochondrial stress and chronic inflammation with characteristic cardiovascular and metabolic alterations, that in sum contribute to a pathophysiological status to be defined as biomolecular allostatic load [24–28].

Several studies demonstrated environmental pollution as a health risk highly relevant for an increased IS incidence [29]. Further, an exposure to a toxic environment such as heavy metals, per evidence, is associated with an increased frequency of non-communicable diseases, such as arterial hypertension and diabetes mellitus with an increased risk of IS [30].

**Fig. 2:**
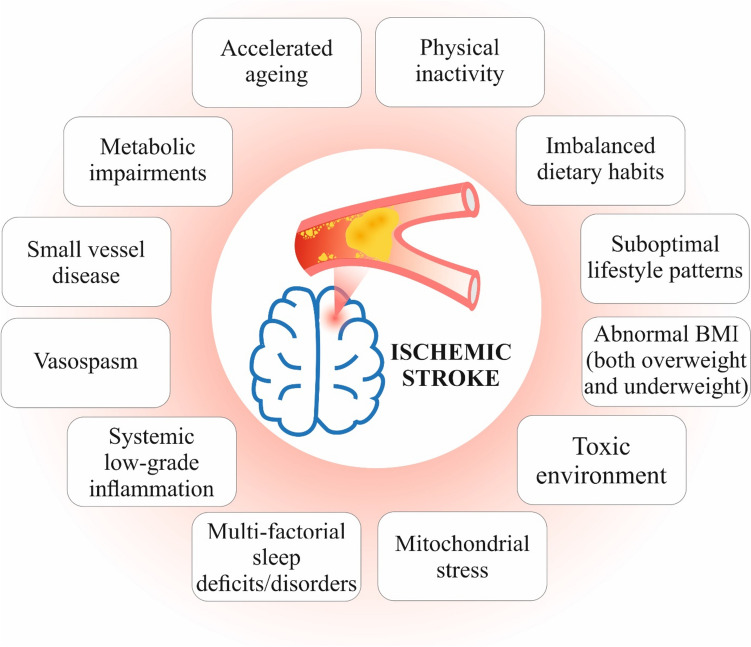
Risk factors of ischemic stroke with sufficient space for cost-effective prevention interventions in primary (suboptimal health) and secondary (clinically manifested collateral disorders contributing to stroke risks) care; these risks are interrelated. For example, sedentary lifestyle and toxic environment both cause mitochondrial stress, systemic low-grade inflammation, and accelerated ageing; inflammageing is a low-grade inflammation associated with accelerated ageing and poor stroke outcomes. Stress overload, decreased mitochondrial bioenergetics, and hypomagnesaemia are associated with systemic vasoconstriction (vasospasm) and ischemic lesions in the brain, e.g. in case of the Flammer syndrome phenotype discussed later on in the article. Imbalanced dietary patterns poor in folate but rich in red and processed meat, refined grains, and sugary beverages are associated with hyperhomocysteinaemia, systemic inflammation, small vessel disease, and increased IS risks. In contrast, Mediterranean diet characterised by strong and systemic anti-inflammatory effects being rich in olive oil, nuts, whole grain, fruits and vegetables, and seafood consumption, but light on dairy and red/processed meat, is known to enhance anti- to pro-inflammatory ratio and to decrease IS risks [33]

Finally, acute and chronic infectious diseases may significantly contribute to IS risks. For example, pulmonary tuberculosis survivors are at significantly higher risk of IS [31]. Moreover, pulmonary tuberculosis patients were demonstrated to develop IS even in absence of conventional vascular risk factors [32] that makes tuberculosis to a very important proof-of-principle model in understanding IS pathomechanisms linked to pulmonary infections and inflammatory pathways. Figure 2 summarises most prominent IS risks with a sufficient space for prevention intervention by the 3PM approach.

## Clinically manifested strokes are only the “tip of the iceberg”: IS with unclear risk factors is a severe challenge to healthcare systems

Worldwide accumulating data indicate that clinically manifested and officially recorded stroke cases do contribute a noticeable portion, which, however, creates a minor share of the entire issue of brain infarction. Rough estimations state that uncovered portion is about 14 times bigger in the population compared to the IS diagnosed portion, which reactive medical approach is applied to [16, 34]. This evident discrepancy is huge dramatically reducing healthcare efficacy and life-quality of populations with significant economic burden to healthcare systems around the world. As early as for the year 1998, the very first evidence-based estimates were published for the USA considering the annual incidence of silent strokes to be at the level of eleven million [35]. Since two decades these levels are continuously increasing.

Being characteristic for the silent brain infarction (SBI), lacunar strokes represent up to 30% of all ischemic strokes. Silent lacunar brain infarctions are either diagnosed by a routine health check-up or by autopsy; frequently these are individuals without stroke history, and brain infarctions discovered are unexpected for them. As the matter of fact, currently reported prevalence of the SBI ranges between 6 and 55% for examined sub/populations depending on the patient stratification criteria and targeted groups under investigation [36–44]. Noteworthy, there is a significant sex-dependent difference in the SBI manifestation, since the silent lesions are more prevalent in females, in contrast to the clinically manifested ischemic strokes which are more typical for males [36, 45–49]. Contextually, the symptomatic interpretation of cerebrovascular diseases and cerebrovascular infarcts is traditionally adapted to the male patient cohorts with the better described clinical picture and more precise recommendations. Therefore, more subtle female-specific symptoms might be wrongly interpreted or even overlooked resulting in poor individual outcomes more frequent for female patient cohorts [50]. Finally, the needs of young populations remain unmet, since SBI-characteristic data for these patient cohorts are underdeveloped [36]. Consequently, an effective protection of young populations against ischemic stroke is lacking in primary care. Considering the tremendous prevalence of IS with unclear risk factors, a robust predictive approach based on individualised health risk assessment and followed by targeted prevention in primary and secondary care is of great clinical utility benefiting millions of high-risk individuals and society at large.

## Key issues to be solved for a cost-effective protection against IS

As notified above, screening towards uncovered SBI cases is expected to result in about 14-times bigger vulnerable subpopulations compared to clinically manifested SBI identified by currently applied reactive medical approach. Specifically, for young sub-populations, SBI-related prevalence is strongly underestimated that significantly hinders targeted protection of youth and young adults in primary care and decreases overall cost-efficacy of IS management. The trend has to be reversed by applying individualised health risk assessment. To this end, an applicability of innovative screening programmes depends to major extent from their effectivity to catch up with vulnerable groups within the stage of reversible damage (focus on suboptimal health, in order to protect individuals against the health-to-disease transition) associated with high cost-efficacy (positive health economy), as well as from the level of acceptance by professionals and in the population. Accompanying educational measures adapted to target groups seem to be the clue.

Per evidence, IS is a multifactorial disease with a highly individual set-up of risk factors which promote the disease development in a synergistic way. Therefore, a holistic diagnostic approach based on individualised patient profiles is essential. Contextually, health risk assessment has to be focused on systemic effects monitored, for example, by natural/physiologic or artificially created (implanted) biosensors. To this end, mitochondria are considered a highly effective multi-functional physiologic biosensor applicable to vulnerable groups in suboptimal health to monitor and to correct their health status protecting against health-to-disease transition [51]. For a non-invasive approach which in many cases reasonably to be performed in a life-long way and certainly highly appreciated by customers, body fluids allowing for monitoring systemic effects are considered highly appropriate. To this end, specifically tear fluid as possessing many advantages (non-invasive holistic approach for monitoring of systemic effects with highly stable biomarkers, amongst others) is extensively under consideration for establishing evidence-based health risk assessment [52–56]. Finally, medical services of high geographic density are decisive to meet urgent needs of populations implementing concepts of predictive diagnostics and targeted protection against preventable disorders [34, 57–59].

## Ongoing 3PM research towards vulnerable groups in the population

### Tear fluid analysis of ischemic stroke patients with multi-factorial risks: preliminary results from international research project

#### Study design

The European proof-of-principle study (Czech Republic–Germany–Austria) aimed to collect and analyse tear fluid samples of stroke patients (16) versus health status well-checked controls (12). Commercially available Schirmer tear strips (sterile diagnostic strips, individually packed, Aivimed GmbH, Wiesbaden, Germany) were used to collect tear fluid samples according to the protocols provided by the manufacturer. After 3 min, the wetting of the Schirmer paper in mm was recorded for both eyes. Immediately after each individual strip was placed into a sterile Eppendorf tube and stored at – 80 °C until mass spectrometry (MS) analysis. MS analysis was performed by Biocrates Life Sciences AG, Innsbruck, Austria, utilising the MxP^R^ Quant 500 kit for metabolomics. The entire analytical procedure is detailed in our previously published article [55]. The experimental metabolomics measurement technique is described by patents EP1897014B1 and EP1875401B1 [60, 61]. The commercially available kit settled by Biocrates Life Sciences was used for qualification and quantification of metabolites classified into 26 different clusters: acylcarnitines, alkaloids, amine oxides, amino acid related, amino acids, bile acids, biogenic amines, carbohydrates and related, carboxylic acids, ceramides, cholesteryl esters, cresols, diglycerides, dihexosylceramides, dihydroceramides, fatty acids, hexosylceramides, hormones and related, indoles and derivatives, lysophosphatidyl-cholines, nucleobases and related, phosphatidyl-cholines, sphingomyelins, triglycerides, trihexosylceramides, vitamins, and cofactors.

#### Metabolic patterns specific for cardio-embolic ischemic stroke (CEIS)

Statistical analysis revealed thirteen metabolic clusters as highly specific for CEIS metabolic shifts (see Table 2). Based on the level of metabolic profile deviation, a “traffic light” system for the health risk assessment has been created, namely, in green colour, metabolic patterns are marked which demonstrate levels similar to controls; in yellow colour, metabolic patterns are marked which up to 2 times are deviated from the control levels; in red colour, metabolic patterns are marked which are either several-times deviated from the control levels or even measurable only in the stroke patients but not in controls (such as xanthine; ceramides (d18:1/25:0), (d18:1/18:1), and (d18:2/24:1); lysophosphatidyl-choline aC26:1; phosphatidyl-choline aeC40:3). Individualised metabolic profiles are exemplified by eight CEIS patients who differ from each other by clinical (BMI, hyperlipidaemia, arterial hypertension, diabetic history) and non-clinical risk factors (age, sex, smoking). Noteworthy, a very important discovery has been made: the more risk factors (clinical, non-clinical, metabolic) are coming together, the younger is the patient e.g. in case of the following:

- 1^st^ patient with CEIS, 50 y.o., no any clinically manifested risk factors, 12 “red” metabolites” and 2 yellow metabolites) *versus*
- 7^th^ patient (35 y.o.), who is a smoker suffering from hyperlipidaemia and demonstrating 28 metabolic parameters in the red area and 5 in the yellow one

**Table 2:**
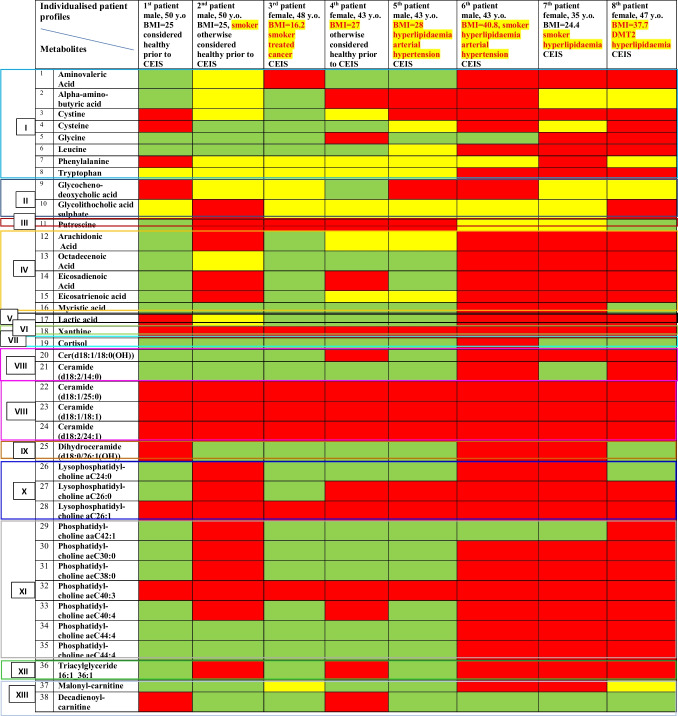
Thirteen metabolic clusters relevant specifically for cardio-embolic ischemic stroke (CEIS) versus healthy controls; Individualised metabolic profiles are exemplified by eight patients who differ from each other by clinical (BMI, hyperlipidemia, arterial hypertension, diabetic history) and non-clinical risk factors (age, sex, smoking); green areas = metabolic patterns which demonstrate levels similar to controls; yellow areas = metabolic patterns which up to 2-times are deviated from the control levels; red areas = metabolic patterns which are either several-times deviated from the control level or measurable only in the stroke patients but not in controls (such as xanthine; ceramides (d18:1/25:0), (d18:1/18:1), (d18:2/24:1); lysophosphatidyl-choline aC26:1; phosphatidyl-choline aeC40:3)

Herewith, we shortly analyse the relevance of the detected metabolites for predictive approach, targeted prevention, and personalised treatments in overall stroke management.

- Cluster I: amino acids and related derivatives

In analytical sets presented here, both proteogenic amino acids/their derivatives (cystine, cysteine, glycine, leucine, phenylalanine, tryptophan) and non-proteogenic amino acids (which the human body obtains from microbiota production, such as 5-amino-valeric acid, and food, such as alpha-amino-butyric acid) were detected in the tear fluid. In consensus, similar patterns including glycine, phenylalanine, and tryptophan which forms a risk score for cardiovascular disease and stroke were reported for blood analysis performed [62–64]. Identification of 5-amino-valeric acid (5-AVA) in relation to the CEIS is an important indication that microbiome profiles are crucial for considering CEIS risks which is instrumental for health risk assessment, predictive diagnostics, and targeted treatments. To this end, pre-clinical research utilising rat model revealed an involvement of 5-AVA in pathogenesis of cardiovascular disease [65].

- Cluster II: bile acids

Alterations in bile acid metabolism were demonstrated as associated with atherosclerosis and inflammation, which are known risk factors of cardiovascular and cerebrovascular diseases [66]. Specifically metabolism of glycochenodeoxycholic acid and glycolithocholic acid sulphate detected in our analytical sets involves complex interactions between the liver, intestine, and gut microbiota, which are known to be involved into multi-factorial risks of CEIS. Further, an increased blood level of glycochenodeoxycholic acid was observed in young patients diagnosed with IS compared to healthy controls [67] which is instrumental for innovative screening programmes focused on healthcare needs of young populations.

- Cluster III: biogenic amines

A panel of biogenic amines was described as a urine biomarker candidate for early diagnosis and prognosis of stroke [68]. Specific for our analytical sets, putrescine significantly increased in CEIS was also identified by Lee et al. (2022) in blood as a novel biomarker specific for the large artery atherosclerosis stroke [69]. Putrescine is involved into neuronal cell damage associated with various type of excitatory neurotoxicity, and the hippocampal putrescine level is increased by global ischemia but is inhibited by L-deprenyl treatment [70] which is instrumental for cost-effective targeted prevention.

- Cluster IV: fatty acids

The central role of fatty acids in the stroke pathomechanisms is evident by in-depth research performed over decades [71]. Specifically saturated fatty acids are involved into atherosclerosis formation. Similarly to our analytical sets, arachidonic acid, octadecenoic acid, and myristic acid patterns were elevated also in blood of IS patients as described by other research groups [72, 73] which is instrumental for health risk assessment and treatment algorithms tailored to individualised patient profiles.

- Cluster V: carboxylic acids

Of the large cluster of carboxylic acids, lactic acid was detected in our analytical sets as highly relevant for CEIS. To this end, lactic acid is a highly sensitive metabolic indicator of the injury progression in cerebral ischemia [74], and corresponding metabolic acidosis is well described as significantly increasing major cardiovascular risks with severe consequences including sudden heart attack and stroke [75] which is instrumental for health risk assessment and cost-effective targeted prevention in general population.

- Cluster VI: nucleobases

In our analytical sets, specifically high xanthine levels were strongly associated with CEIS. Xanthine is a product of the purine degradation pathway subsequently converted to uric acid by the xanthine oxidase. Noteworthy, increased xanthine levels in the cerebrospinal fluid are considered a predictor of IS lethal outcomes [76]. Corresponding pathomechanisms involve xanthine into severe oxidative stress by converting xanthine to uric acid and generating reactive oxygen species (ROS) as a by-product that is directly associated with high IS risks [77] which is instrumental for predictive and prognostic approach in primary and secondary care.

- Cluster VII: hormones

The stress-associated hormone cortisol was detected in one of the severe cases of CEIS with particularly poor outcome. In consensus, an increased serum cortisol level is considered a marker of severity, short- and long-term prognosis, and mortality after acute IS [78].

- Clusters VIII and IX: ceramides and derivatives

Circulating ceramide levels are considered powerful predictors upstream to severe cardiovascular events later on, such as myocardial infarction and stroke. Ceramides containing the C16, C18, and C24:1 acyl chains demonstrate a superior independent predictive value for plaque instability and/or future fatality than conventional lipid profile measures, including LDL cholesterol [16, 79–83]. Still, there are very few studies which have investigated individual fractions of ceramides in the blood associated with IS risks and mortality [84].

- Clusters X and XI: lysophosphatidyl-cholines and derivatives

Lysophosphatidyl-cholines (LPC) are bioactive lipids derived from phosphatidylcholines. LPC also serve as signal molecules released by apoptotic cells to attract phagocytes being, therewith directly linked to inflammatory disorders [85]. Corresponding mechanisms are complex and vary significantly from case to case, depending on the multi-factorial context ranging from damaging effects (e.g. via endothelial dysfunction) leading to IS development, to regeneration processes after IS (anti-inflammatory mechanisms) which they are involved in [85–88]. Contextually, individual LPC fractions and derivatives should be carefully considered for individualised patient profiles for predictive molecular sets, health risk assessment, and treatments tailored to the person.

- Cluster XII: triglycerides

Hypertriglyceridemia arises from disruptions in triglyceride metabolism, leading to elevated levels of plasma triglycerides. Elevated plasma triglycerides, especially in non-fasting states, have been described as a risk factor of IS. Well investigated pathomechanisms which hypertriglyceridemia is involved in are atherosclerosis, thrombosis, and elevated blood viscosity [89]. Specifically, the triacylglyceride 16:1_36:1 fraction identified in our analytical sets was reported as being specific (tear fluid) for diabetic patients diagnosed with diabetic retinopathy as a risk factor highly relevant for IS [55] which is instrumental for predictive diagnostic approached followed by cost-effective preventive measures.

- Cluster XIII: acylcarnitines

Acylcarnitines are the key molecules for energy production via β-oxidation by transporting fatty acids to mitochondria, for example, when IS causes an imbalanced energy pathways and deprived glucose supply. Distributions of acylcarnitines in brain affected by IS was studied in animal models demonstrating significant deviation in corresponding metabolic patterns and indicating an importance for quantitative monitoring of acylcarnitines profiles for targeted handling of damage caused by stroke [90]. Finally, decanoylcarnitine and octanoylcarnitine were demonstrated as independent discriminants specifically for the cardioembolic stroke. The levels of decanoylcarnitine and octanoylcarnitine is significantly higher in blood of stroke patients at high risk of cardioembolism compared to low and intermediate risk [91] which is instrumental for health risk assessment followed by cost-effective preventive measures.

### Tear fluid analysis of diabetic patients with retinopathy: making ophthalmologic units to the check-point for personalised IS prediction in diabetes care

“Ut imago est animi voltus sic indices oculi”, “the face is a picture of the mind as the eyes are its interpreter”, has been already declared by Cicero (106–43 BC). Scientists translated and transformed the citation to “the eye is the window to the brain”, and this phrase is more than a philosophical consideration but an anatomical truth. The eye belongs to the central nervous system, and axons of the retinal ganglion cells are bundled to the optic nerve projecting to the optic chiasma, thus into the brain. Therefore, multiple studies evaluated potential ocular signs of CNS diseases from neurodegenerative disease like multiple sclerosis and Alzheimer’s disease to cardiovascular diseases like stroke.

Diabetes mellitus type 2 (DMT2) patients are several times more susceptible to a stroke event due to various cerebrovascular complications [92]. Micro- and macro-vascular changes characteristic for DM lead to particularly poor IS outcomes, increased mortality, and poor post-procedural quality of life [93, 94]. Moreover, glucose-lowering therapies are insufficient protection against IS risks in DM [95, 96]. DM and stroke are synergistically involved in the induction of cerebral-cardiac syndrome (CCS) characterized by arrhythmias, myocardial damage, and heart failure: DM drives chronic inflammation which aggravates CCS. Corresponding pathomechanisms involve inflammasomes [97]. IS has emerged as a serious complication of inflammation and cytokine storm by the severe acute respiratory syndrome coronavirus 2 (SARS-CoV-2) [98]. To this end, COVID-19-related IS with particularly high rates and poor outcomes was recorded for patients with diabetes, hypertension, and hyperlipidaemia [99, 100]. Contextually, primary prevention of DMT2 is the most cost-effective approach against DM-associated IS with particularly poor outcomes.

**Fig. 3:**
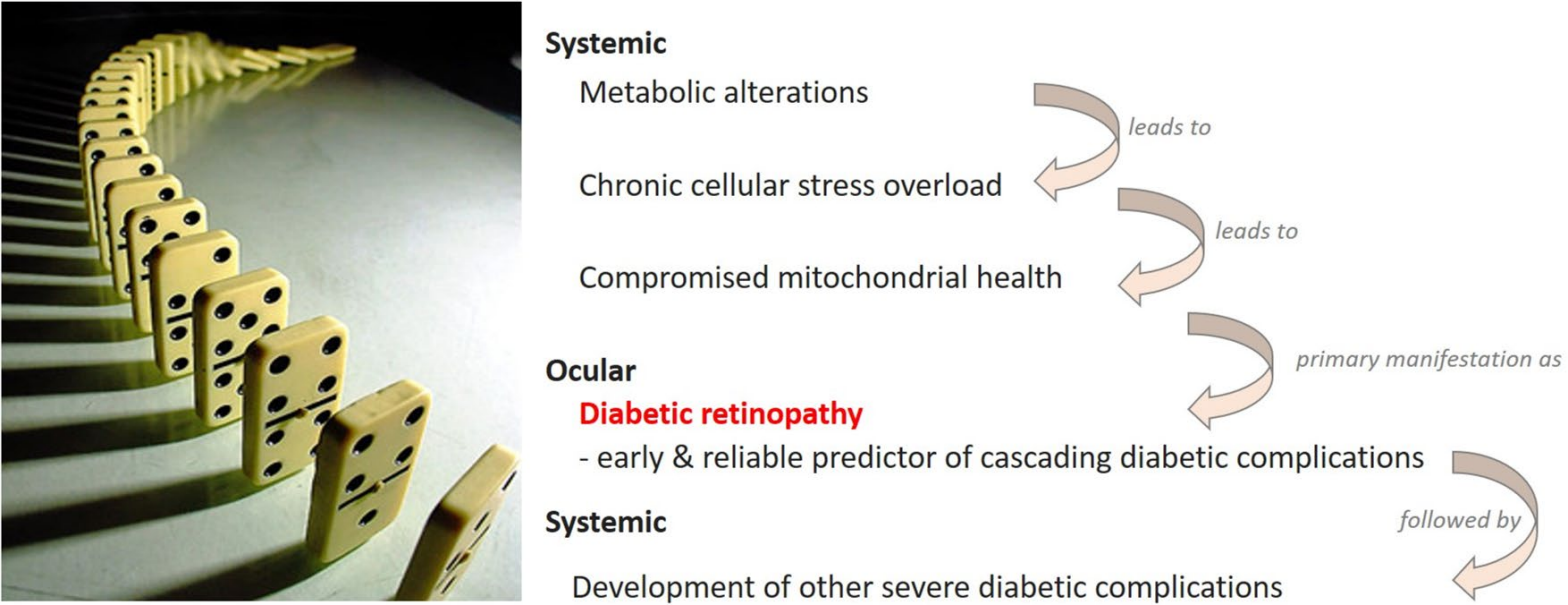
DM-associated complications cascade appears as a “domino effect” downstream to proliferative DR characteristic for patients predisposed to severe comorbidities such as ischemic stroke; the image is adapted from [102]

Diabetic retinopathy (DR) is a microvascular disease of the retina caused by systemic damaging effects triggered by metabolic disorders (diabetes mellitus, DM types 1 and 2). DR is clinically manifested as a progressing damage of retinal capillaries with secondary visual impairment widely recognised as the leading cause of blindness in the working-age population [101]. By far, not every DM patient develops DR and other secondary complications. However, once manifested, DR acts as an early indicator of complications related to DM. To this end, specifically proliferative DR is considered an independent predictor of cascading DM-related complications appearing as a “domino effect” as demonstrated in Fig. 3 [102].

An accumulated scientific evidence demonstrates the clinical manifestation of DM as a significant risk factor for stroke predisposition: patients with DM-history are at 2 to 4 times higher stroke risk compared to individuals without DM history. Moreover, the stroke risk severity correlates well with an increasing DR stage and disease progression [103]. Consequently, the prediction of DR progression in DM patients is highly instrumental for the cost-effective secondary care of the affected patient cohort with the potential to protect them against DM-promoted stroke manifestation. This concept has been implemented in the EPMA-promoted European project dedicated to biomarker-panels specific for the progressive DR which can be potentially identified in the tear fluid of affected individuals [55].

**Fig. 4:**
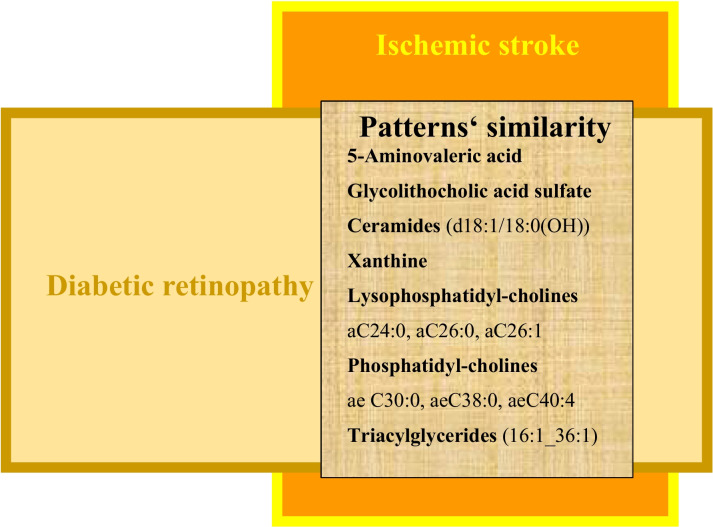
Pattern’s similarity in the tear fluid detected in the vulnerable group of DR patients and patients with clinically manifested cardio-embolic ischemic stroke

Indeed, preliminary data resulted from the study demonstrate a unique metabolic signature which is characteristic for proliferative retinopathy and can be routinely detected by applying mass spectrometric analysis to the tear fluid taken from vulnerable individuals. Furthermore, a great discovery has been made towards pattern’s similarity between both patient cohorts, namely, patients with diabetic retinopathy as a group at risk for stroke development and patients with clinically manifested ischemic stroke as imaged in Fig. 4. To this end, the implementation of a 3PM approach analysing the tear fluid of diabetic patients can identify the combined risk for diabetic retinopathy and IS with below presented metabolites as potential biomarker-sets.

- 5-Aminovaleric acid

Glucose and amino acid metabolism are closely related [104]. We found 5-aminovaleric acid and betaine distinguishable specifically in the tear fluid of the DR patients [55]. A similar association with cardiovascular morbidity and mortality has been found in the gut microbiome [105].

- Glycolithocholic acid sulphate

Bile acids are critical factors in regulation of glucose, energy, and lipid associated metabolic pathways. In this context, a disturbed bile acid homeostasis has been linked to metabolic diseases like obesity, dyslipidaemia, and DM type 2 [106], which are well-known main risk factors for cardiovascular disease and stroke. In our study, in which the metabolic profile of diabetic patients was determined in tears, particularly the glycolithocholic acid sulphate, a molecule that is part of the bile acid metabolic cluster could only be detected in DR patients [55]. Moreover, regulation of bile acid and the gut microbiome have been proposed as predictive biomarkers, protective factors, and treatment targets for diabetes, atherosclerosis, and IS [107–109].

- Ceramides

We found ceramide fractions specific for patients affected by diabetic retinopathy [55]. This result is consistent with the implication of lipotoxic ceramides in inflammation, β-cell apoptosis, and insulin resistance; moreover, they are involved in inducing and promoting microvascular and macrovascular complications relevant for DM and cardiovascular disease [110–112]. In vitro experiments could show that amongst others, ceramides disrupt vasodilation [113]. Thus, ceramides are considered reliable biomarkers for DM and cardiovascular disease, and its inhibitors are potential treatment targets in preventing DM and IS.

- Xanthine

Xanthine was found in tears of DR patients [55]. The intermediate product of the purine nucleotide catabolism can bind in vitro to all nucleotide bases and normally does not incorporates into DNA and RNA [114]. Oxidative and nitrosative stress, however, can lead to oxidative deamination of DNA and the build of harmful deoxyxanthosine [114]. Both, oxidative and nitrosative stress, play a well-known key role in pathogenesis of DM type 2 and cardiovascular disease responsible for cellular stress, mitochondrial dysfunction, metabolic syndrome, inflammation, and apoptosis [115].

- Lysophosphatidyl-cholines (LPC)

Highly increased LPC are correlated to neurodegeneration and diabetic retinopathy [87]. We found LPC profiles from diabetic retinopathy patients different to healthy controls [55]. Studies found increased level of LPC involved in blood-retinal-barrier damage, oxidative stress, and induction of vasopermeability [116, 117]. Similarly, Yuan et al. provoked demyelination in cerebellar organ culture treated with LPC that could be recovered by co-culture with regulatory T cells as potential treatment target after stroke [118].

- Phosphatidyl-cholines

Phosphatidyl-cholines are components of plasma lipoproteins regulating circulating lipoprotein levels such as low-density (VLDLs) and high density (HDL) lipoproteins ^113^. Consequently, several studies demonstrated phosphatidyl-cholines related to a decreased risk for diabetes, cardiovascular disease and stroke [119–121].

- Triacylglycerides

Though the exact role of distinct lipid classes in DM and stroke is still unknown, it is widely accepted that lipids and lipoproteins significantly contribute to their pathogenesis. We found triglycerides profiles measured in the tear fluid specific for the diabetic proliferative retinopathy [55]. Consistent with this data, an association between hypertriglyceridemia in diabetic patients and lower-extremity amputation risk has been shown [122]; blood triglycerides have been demonstrated as reliable pre/diabetes biomarkers [123]. In parallel, specific lipid profiles were demonstrated to be linked to high-risk for ischemic stroke [121].

### Silent brain infarction (SBI) as an independent indicator of an active health‑to‑disease transition: making GP practice to the check-point for cost-effective IS protection in primary care.

“Silent strokes are not so silent” as stated by experts [16] who deal daily with abnormal brain scans looking for subtle changes indicated by SBI. Indeed, manifested SBI strongly predicts extended patterns of silent infarction zones [124] acting therefore as a powerful predictor of ischemic stroke in affected individuals. To this end, several research groups demonstrated over 50% prevalence of SBI in the ischemic stroke patients [125]. Moreover, an intensity of cognitive impairment and dementia correlates with SBI extend [126–129]. Similarly, in a Japanese study dedicated to the cognitive impairments of Alzheimer’s disease patients, every 3rd patient was co-diagnosed with SBI [130]. Specifically when localised in the thalamus, SBI causes impaired short-term memory performance [131–133], and non-thalamic SBI impairs psychomotor speed task performance [125]. SBI are frequently reported for people diagnosed with major depression [125, 134–136] and bipolar disorder type II with a suicide attempt [137]. Further, there is an association between migraine attacks and SBI manifestation [138, 139]. Due to high oestrogen levels, the female sex is a well-acknowledged risk factor of migraine with aura, thromboembolism, ischemia-induced aura, and ischemic stroke [140]. Female sex and migraine with aura are the characteristics for the Flammer syndrome phenotype (FSP, below is a case report presented), the affected carriers of which are strongly predisposed to SBI and normal-tension glaucoma (NTG) [141, 142].

#### Flammer syndrome phenotype (FSP) carriers are predisposed to silent lacunar brain lesions: a prominent clinically relevant example for the cost-effective primary protection against IS.

FSP is a well-described clinically relevant suboptimal health condition [143] strongly predisposed to disorders related to non-compensated stress overload such as pregnancy-associated complications, normal-tension glaucoma, lacunar ischemic lesions, and aggressive metastatic cancers [16, 144–149]. Corresponding pathomechanisms include primary vasospasm and low blood pressure, shifted circadian rhythms and sleep patterns, shifted sense regulation patterns (e.g. reduced thirst perception and thermal dysregulation), psychosomatic particularities (e.g. perfectionism), and compromised mitochondrial health, amongst others [144]. Corresponding systemic effects are well reflected in altered molecular patterns recorded in the blood of FSP carriers [150–152].

Herewith, we introduce the case of a 59-year-old woman who is a FSP carrier in suboptimal health condition. A specialised survey revealed strongly pronounced signs and symptoms of FSP including low body mass index, low blood pressure, migraine with aura, and strong vasospastic reactions under stress conditions; high stress-sensitivity further aggravated by meticulous personality and permanent work/stress overload caused by a highly ambitious international academic career of the patient.

**Fig. 5:**
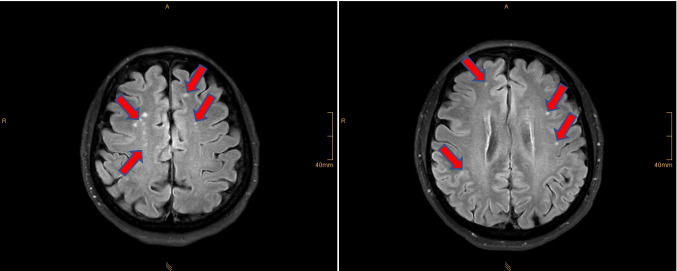
Brain-MRI; the multi-site pattern of lacunar microinfarction zones is clearly visible in the image (red arrows); 3Tesla-MRI was performed at the radiological clinic, University of Bonn, Germany; weight-adjusted intravenous contrast medium administration (CMA, gadolinium) was used

Objectively diagnosed and measured are as follows:

A. Family history of ischemic stroke (both parents with a successful academic career and high education level in medicine).
B. Significantly increased endothelin-1 level (3.2 pg/ml) in blood serum, retinal ischemic lesions diagnosed early in life, and connective tissue impairments.
C. Brain MRI revealed silent lacunar ischemic lesions (see Fig. 5).

## Protection against sudden cardiac arrest/death as a positive side effect benefiting populations by 3PM approach in IS primary care

An unexpected loss of cardi-vascular function resulting in circulatory collapse and death is defined as sudden cardiac death (SCD) as the end result of the sudden cardiac arrest (SCA) associated with about 90% mortality and high functional/cognitive disability of survivors [153]. Approximately 2 million cases of SCD are registered annually worldwide [153, 154]. Although the SCA incidence is relatively low in the general population, this medical condition is considered most unpredictable and demands robust health risk assessment for timely prediction and targeted prevention. To this end, a high level of similarity between risk factors of IS and SCA is instrumental for advanced health risk assessment to be implemented for innovative population screening saving lives and money. Table 3 summarises available data considering the most prominent demographic, socio-economic, non-clinical, and clinical risks.

**Table 3:** Significant similarity between risk factors of IS and SCA: demographic, anthropometric, socio-economic, clinical, and non-clinical factors are considered. Abbreviations: *IS* ischemic stroke, *SCA* sudden cardiac arrest, *Hhcy* hyperhomocysteinaemia

Health risk factors	IS	SCA	Comments	Scientific literature
Demographic				
Progressing age	Yes	Yes	Although elderly is an acknowledged risk for both IS and SCA, IS incidence is particularly increasing in young populations during last decade	Abundantly available
Male sex	Yes	Yes	Female sex is frequently associated with poorer outcomes, since symptoms are adapted to males	Abundantly available
Anthropometric				
Abnormal BMI	Yes	Yes	Both underweight and overweight are risks for IS and SCA, but the so-called obesity paradox is described as being potentially protective against poor IS outcomes, concluding that not BMI itself but rather shifted metabolism may be risky	[16, 17, 20, 154–156]
Central adiposity (waist-to-hip ratio)	Yes	Yes	Systemic effects	[154, 157]
Socio-economic				
- Low education - Low income - Career-dedicated individuals (meticulous personality)	Yes	Yes	“Blue collar-workers” are considered a vulnerable socio-economic category. On the other hand, “white collar-workers” demonstrate category-specific vulnerability as well	[16, 154]
Clinical				
Cardiovascular susceptibility (in general)	Yes	Yes	Including primary vasospasm (e.g. in Flammer syndrome phenotype susceptible to silent brain lesions, lacunar iIS)	Abundantly available [16]
Arterial hypertension				
Psychosomatic and psychiatric conditions	Yes	Yes	- Pre-existing psychiatric conditions - Psychotropic medication - Meticulous personality (e.g. Flammer syndrome)	[16, 154]
Short sleep, sleep apnoea, poor sleep quality	Yes	Yes	A longer sleep duration (< 9 h a day) protects from SCA	Abundantly available [158]
Chronic pain (CP)	Yes	Yes	Strong association between chronic low back pain, incident transient ischemic attack and stroke. Further, CP-medication increases risks of potentially fatal ventricular arrhythmia	[159, 160]
Systemic inflammation	Yes	Yes	Both microflora-induced and sterile inflammation with predictive and prognostic power	Abundantly available
Asthma	Yes	Yes	Asthma is associated significantly with increased risk of both IS and SCA	[161–163]
Metabolic shifts and impairments	Yes	Yes	- Hyperglycaemia - Hypoglycaemia - Hypokalaemia - Hypomagnesemia - Hyperhomocysteinaemia	Abundantly available
Mitochondrial homeostasis and signalling	Yes	Yes	Mitophagy is an excellent target for prediction, therapy and prognosis of both IS and SCA	Abundantly available
Trauma	Yes	Yes	Highly individual outcomes	Abundantly available
Treated and active malignancy	Yes	Yes	Systemic effects	Abundantly available
Drug overdose	Yes	Yes	Systemic effects	Abundantly available
Epilepsy	Yes	Yes	Reciprocity of mechanisms	Abundantly available
Sepsis	Yes	Yes	Systemic effects	Abundantly available
Non-clinical				
Hyperthermia	Yes	Yes	Systemic effects	Abundantly available
Dehydration	Yes	Yes	Systemic effects	Abundantly available
Asphyxia/hypoxia	Yes	Yes	Systemic effects	Abundantly available
Physical inactivity/overload	yes	Yes	Systemic effects	Abundantly available
Imbalanced diet	Yes	Yes	Systemic effects	Abundantly available
Stress overload	Yes	Yes	Systemic effects	Abundantly available
Smoking	Yes	Yes	Systemic effects	Abundantly available
Abnormal alcohol intake	Yes	Yes	Systemic effects	Abundantly available
Malnutrition	Yes	Yes	Systemic effects	Abundantly available
Toxic environment	Yes	Yes	Systemic effects	Abundantly available

### Case report

A male patient, 72 years of age, underwent investigations utilising a questionnaire and multi-omic analysis (metabolites’ profiling and mitophagy quantification) of his tear fluid three months before a sudden heart arrest/death which happened to him rather unexpectedly according to comments of his medical doctor in charge.

Below, we report on multi-factorial risks which are well in consensus with the data presented in Table 3. Moreover, according to metabolic analysis performed in the tear fluid, 10 biomarkers correspond to the molecular patterns relevant specifically for cardio-embolic ischemic stroke (clusters I, IV, V, VIII).

- Demographic risks: male, 72 y.o.
- Socio-economic risks: blue collar-worker, low level of education, low income—all socio-economic risks
- Clinical risks: treated arterial hypertension; BMI = 28; higher waist-to-hip ratio; short sleep (6 h daily), snoring; since 5 years daily intake of pain-killers due to orthopaedic medical conditions (chronic pain/ inflammation).
- Non-clinical risks: severe smoker, red meat-rich diet
- Risky metabolic patterns in the tear fluid (see Section "Tear fluid analysis of ischemic stroke patients with multi-factorial risks – preliminary results from international research project", Table 3)
  - Cluster I: alpha-amino-butyric acid (4 times higher, red area), cysteine (3 times higher, red area), cysteine (1.5 times higher, yellow area), leucine (2 times higher, yellow area), and phenylalanine (2 times higher, yellow area) against the average value in the control group
  - Cluster IV: arachidonic acid (4 times higher, red area), octadecenoic acid (2 times higher, yellow area), and eicosatrienoic acid (4 times higher, red area) against the average value in the control group
  - Cluster V: lactic acid (3 times higher, red area) against the average value in the control group
  - Cluster XIII: malonyl-carnitine (3 times higher, red area) against the average value in the control group.

Finally, quantitative PCR analysis of cf-mtDNA demonstrated mitochondrial burnout by extremely low levels of mitophagy measured in the tear fluid and compared with the age-matched reference data. The mitophagy quantification methodology in a liquid biopsy (blood) is described elsewhere such as [164] and further adapted to the tear fluid analysis followed by individualised data interpretation (the know-how of “3PMedicon GmbH” performing internationally validated tests [165]).

## Expert recommendations for multi-professional consideration

### 3PM innovation to advance health risk assessment and protection against IS

Keeping in mind the above presented research data, for the follow-up studies, we state herewith that a number of risk factors are currently insufficiently considered which synergistically may predispose individuals at any age to IS development. To this end, a contribution specifically to young IS can be expected by below listed medical conditions which are to great extent unexplored but earned a strong indication in this paper.

1. Vascular stiffness of heterogeneous causality and primary vasospasm particularly in young individuals without traditional IS risks [16, 166].
2. Brain tissue remodelling (e.g. early in life mild and severe pre- and perinatal asphyxia [58].
3. Reciprocity between IS and cancers [16].
4. Mitochondrial stress reflected in condition-specific homeostasis patterns (see below Section “Mitochondria as a vital biosensor and attractive therapeutic target”).
5. Microbiota-gut-brain related inflammageing (see below Section" Microbiota-gut-brain axis: A promising intervention by pro- and prebiotics").
6. Specific psycho-somatic conditions: according to the biopsychosocial model of health and disease, psychological functioning is a crucial but rather neglected factor to be further used for the patient stratification. Specifically with a focus on stress coping capabilities, innovative biomarker sets are of pivotal importance to assess biopsychosocial profiles towards healthy aging in a society constantly getting older.
7. Accelerated ageing of heterogeneous causality (e.g. unhealthy lifestyle, suboptimal dietary habits, stress overload, toxic environment, metabolic syndrome, sleep disorders).

Corresponding risks can be estimated individually applying specialised surveys (to be further developed) at the level of primary care (general practitioners; see Section "General practitioners at the forefront of individualised IS prevention: preventable risks and reversible health-to-disease transition are in focus") and secondary care (e.g. ophthalmologic units; see Section "Tear fluid analysis of diabetic patients with retinopathy: making ophthalmologic units to the check-point for personalised IS prediction in diabetes care") followed by the tear fluid analysis of validated targets.

Considering the reciprocity between IS and cancers (above point 3), IS and cancers share several risk factors and pathomechanisms including oxidative stress, vascular impairments (including primary and secondary vasospasm), compromised mitochondrial health, and inflammation, which synergistically cause a vicious circle of the key systemic effects, namely, ischemia-reperfusions, ischemic lesions, blood–brain barrier breakdown, extensive tissue remodelling by the core of metalloproteinases, and pre-metastatic niches [16]. Further, cancer therapies can lead to an increased risk of IS: Meta-analysis shows that tamoxifen, a synthetic chemo-preventive drug in breast cancer management, increases ischemic stroke risk (82%) in women diagnosed with breast cancer [167]. Similarly, the use of aromatase inhibitors leads to a higher risk of IS and myocardial infarction in individuals diagnosed with breast cancer. Contextually, individualised patient profile is instrumental for IS-associated health risk assessment to advance overall management in cancer affected and cancer predisposed individuals.

Preliminary data considering molecular patterns in the tear fluid with a significant predictive power towards IS predisposition are presented in this paper (see Sections "Tear fluid analysis of ischemic stroke patients with multi-factorial risks – preliminary results from international research project" and "Tear fluid analysis of diabetic patients with retinopathy: making ophthalmologic units to the check-point for personalised IS prediction in diabetes care"). In terms of causality, several molecular pathways demonstrate an important role in and high specificity for some or several above listed medical conditions. For examples, there is an evident overlap between molecular patterns involved in IS and diabetic retinopathy as an early indicator of IS risk in DM as demonstrated in Fig. 4. Just to exemplify some of them such as the 5-aminolevulinic acid/pathway, which are also characteristic for an altered mitophagy [168], insomnia [169], stress regulation [170], and modulation of microbiota-gut-brain crosstalk [171, 172], amongst others. Further, ceramides are considered mediators of oxidative stress and inflammation in cardiometabolic disease [173], negatively affecting mitochondrial respiratory chain function and fission/fusion activity [174], altered sleep–wake behaviour [175], vascular stiffness, and remodelling [176], amongst others. Xanthine/pathway regulation is involved in mitochondrial homeostasis and stress-driven anxiety-like behaviour [177] as well as molecular mechanisms of arterial stiffness [178]. In order to assess individual health risks, an application of machine learning/artificial intelligence is unavoidable for an accurate data interpretation performed by the multiparametric analysis (see Section "Application of artificial intelligence to the 3PM approach").

### Mitochondria as a vital biosensor and attractive therapeutic target

Mitochondria, acting as the “powerhouse”, routes the key events in human cells including proliferation, differentiation, growth and death, and modulate systemic effects such as stress response, redox balance, and severity of the acute and chronic disorders, amongst others. The reciprocity between mitochondrial and organismal health status has been demonstrated: on one hand, compromised mitochondrial health causes systemic damage, and on the other hand, organismal health-to-disease transition is reflected in mitochondrial dysfunction. To this end, mitochondrial population size, fission, fusion, biogenesis, and mitophagy are measurable parameters of mitochondrial homeostasis and, therefore, instrumental for predictive diagnostics and targeted treatments. Contextually, holistic 3PM strategies consider mitochondria as natural health status biosensor and an attractive therapeutic target in primary and secondary care. Table 5 presents conditions with elevated IS risk and mitochondrial component as an attractive target for 3PM strategies in primary and secondary care [179].

**Table 4:** Conditions relevant for IS risks with mechanisms involving mitochondria as a natural vital biosensor [179]

*Conditions*	*Prominent examples and clarifying notes*
Environmental and professional occupation risks with adverse effects on mitochondrial health status relevant for IS management in primary and secondary care	
Exposure to air pressure drop and elevation	Professional occupation; frequent flights
Toxic environment	Ambient air pollution, amongst others
Shift work	Changing physiologic circadian rhythms
Time-frame of daily activities deviating from individual circadian rhythm	Circadian rhythm associated sub/cellular mechanisms
Socio-economic and life-style associated risks relevant for mitochondrial impairments and IS	
Malnutrition and suboptimal dietary habits	Body weight gain and lost, shifted metabolism
Suboptimal lifestyle	Multifactorial risks including imbalanced stress overload and sedentary life-style, amongst others
Genetic risks relevant for mitochondrial impairments and IS	
Family predisposition to severe pathologies	Inborn disorders and genetic predisposition, e.g. to CVDs, autoimmune disorders, and stroke
Medical conditions highly relevant for mitochondrial impairments and IS predisposition	
Suboptimal health conditions	Health-to-disease transition linked to decreasing mitochondrial health quality
Vascular status associated conditions	Abnormally low and high blood pressure, vascular stiffness, increased endothelin-1 level, primary and secondary vasospasm, subtle but chronic ischemia–reperfusion, for example, typical for individuals with Flammer syndrome phenotype
Psychologic distress, psycho-trauma, and post-traumatic stress disorder	Mitochondrial stress potentially leading to mitochondrial burnout
Chronic fatigue and burnout syndrome	
Sleep disorders	
Chronic inflammation and associated conditions	Mitochondrial impairments to be diagnosed and treated
Prolonged and impaired wound healing	
Microbiome shifts/dysbiosis	Mitochondrial stress caused by bio-toxins; relevant sources of information: body fluids, skin, oral and vaginal cavities, digestive and urogenital tracts, airways, wounds, etc
Allergies	Breathing-related ischemia, mitochondrial stress
Asthma	
Premature ageing	Of multi-factorial origin progressed biologic age against chronologic one associated with decreased mitochondrial functionality
Medications highly relevant for mitochondrial impairments and IS predisposition	
Complex treatment of multi-factorial diseases	Mitochondrial component has to be considered in secondary prevention, monitoring of the therapy efficacy and predisposition to IS such as aggressive treatment of malignancies

### Microbiota-gut-brain axis: a promising intervention by pro- and prebiotics

Microbiota-gut-brain axis plays the central role in brain pathologies, ageing related systemic inflammation (inflammageing), premature ageing, and ageing-associated disorders with poor outcomes. To this end, the reciprocity between acute ischaemic stroke and gut dysbiosis has been clearly demonstrated: Ischaemic stroke is associated with acute gut microbiome dysbiosis indicated by prevalent Enterobacteriaceae which in turn exacerbates brain infarction [180]. Further, experimental stroke (middle cerebral artery occlusion) in young and aged male mice demonstrated microbiota-dependent outcomes. Dysbiosis characteristic for poor microbiota in aged mice, if transplanted to young mice, increased their mortality after induced stroke, thereby decreasing quality of behavioural patterns and increasing overall cytokines’ level [181]. In the same experimental sets, microbiota rejuvenation in aged mice significantly improved their outcomes after induced stroke. Contextually, pathogenesis of stroke involves imbalanced intestinal microbiota with strong pro-inflammatory response. Therefore, preventive probiotics and prebiotics application is considered a promising therapeutic intervention with significant health benefits to vulnerable subpopulations. Probiotics are defined as “live strains of strictly selected microorganisms which, when administered in adequate amounts, confer a health benefit on the host”, whereas prebiotics are defined as “a substrate that is selectively utilized by host microorganisms conferring a health benefit” [182]. These natural food supplements demonstrate evidenced properties to mitigate IS risks through several mechanisms modulating immune response and microflora composition with strong systemic effects [183] instrumental for predictive and preventive approach in overall IS management.

### Application of artificial intelligence to the 3PM approach

As argued above, IS is a multi-factorial disease. Contextually, a robust health risk assessment is pivotal for 3PM innovation, and data-driven AI/machine learning (ML) techniques are required [184]. Generally speaking, ML is a multi-parametric statistical model adapted to the data available; this “learning” process results in a “pattern” or “rule” characteristic for the medical condition under consideration. ML can be supervised or unsupervised. In a supervised learning, the model is trained based on datasets coupled with known clinical outcomes such as IS manifestation and severity grade. In the next step, based on the established model health risk assessment/prediction/prognosis have to be validated by application of algorithms to a new patient cohort which was not part of the training round. ML models are particularly applicable to cases, when there is no any “decisive” biomarker capable to rigorously discriminate between “yes” and “no” disease. Noteworthy, opportunities for ML go significantly beyond the disease classification covering a possibility to predict disease development, progression and severity. Opposed to supervised learning, unsupervised learning aims at inferring patterns from data without having access to a label, such as phenotype. Consequently, ML models can discard features which are irrelevant for the prediction (sparse models). The set of features selected by the algorithm can then establish a precise biomarker signature which is characteristic for the medical condition under consideration [185–187]. However, any ML model is capable to capture a pattern involving the data which the algorithm was originally trained on. Therefore, the next step is thus to integrate data (coming from the same patient) across all relevant biological scales and then to develop more advanced AI/ML models on these combined data sources. Single organizations typically do not possess or are unable to collect such multi-modal data in sufficient quantity and representativeness of the entire disease population. Hence, it will be necessary to set up an according data ecosystem across multiple international institutions. Given the current legal constraints on sharing of patient-level data in many areas of the world (such as the GDPR in the European Union), such an effort can only be successful, if data can be accessed and used in a decentralized manner, for example, with the help of federated data access and federated machine learning concepts, including swarm learning [188]. Noteworthy, implementation of such concepts is technically, legally, and organisationally challenging, and requires a close collaboration between multi-professional groups including physicians, data scientists, software engineers, lawyers, patient organisations, and policy-makers.

### General practitioners at the forefront of individualised IS prevention: preventable risks and reversible health-to-disease transition are in focus

The majority of IS cases are preventable, and modifiable risk factors are known. Contextually, the paradigm in overall IS management has to be shifted from reactive care of clinically manifested pathology to prediction, targeted prevention, and personalised treatment of suboptimal health conditions characterised by a reversible damage to health. Therefore, specifically general practitioners are at the forefront implementing the paradigm of 3P medicine in targeted prevention of IS. For doing that, predictive diagnostic tools are developed to a great extent as highlighted above. What might be the practicable workflow in the overall procedure and what are the remaining bottle-necks?

A. Phenotyping based on specialised surveys

Highly relevant information can be received by routed interviews performed at regular check-up including family predisposition, health status, lifestyle habits, sleep and dietary patterns, and anthropometrics, amongst others—all highly relevant for preliminary health risk assessment followed by in-depth genotyping.

B. In-depth molecular analysis based on liquid biopsy

This tool is instrumental for predictive diagnostics based on molecular patterns specific for disease pre-stages. Section "Tear fluid analysis of ischemic stroke patients with multi-factorial risks – preliminary results from international research project" provides an example on how non-invasive tear fluid analysis can be of great clinical utility to predict IS development in individuals with and without clinically evident IS predisposition. Follow-up studies are essential to finalise validation of proposed biomarker-patterns.

C. Health risk assessment based on individualised patient profiles

A combination of (A) and (B) comprises a set of parameters for an individualised risk assessment. Due to precise multi-parametric analysis, an involvement of artificial intelligence (AI) is essential to create algorithms which allow for highly precise predictive diagnosis, evaluation of modifiable risks, and elaboration of programmes for targeted mitigation measures. The AI aspects are further detailed in the below presented Section "Application of artificial intelligence to the 3PM approach".

D. Multi-professional collaboration in 3P medicine

A tight collaboration between all relevant stakeholders is essential including healthcare industry, researchers, educators, patient organisations, and policymakers, amongst others. Effective realisation of 3PM principles, strategies, and approaches saves human and financial resources. However, 3PM innovation should be supported administratively and economically at any stage “from bench to bedside”.

E. Educational programmes in 3P medicine is a “must” for the society

In order to implement 3PM innovation, the essential pillars are as follows: broad acceptance in the population, patient-doctor cooperation (participatory medicine), and high level of professionalism at the side of caregivers. This is challenging and can be achieved by introducing specialised educational programmes for professionals and general population utilising high level of 3PM didactic materials [189].

## Data Availability

Not applicable.

## Abbreviations

AI: Artificial intelligence
5-AVA: 5-Amino-valeric acid
BMI: Body mass index
CEIS: Cardio-embolic ischemic stroke
cf-mtDNA: Cell-free mitochondrial DNA
CP: Chronic pain
DALYs: Disability-adjusted life-years
DM: Diabetes mellitus
DMT2: Diabetes mellitus type 2
EPMA: European Association for Predictive, Preventive and Personalised Medicine
FSP: Flammer syndrome phenotype
GBD: Global burden of disease
GDPR: General data protection regulation
Hhcy: Hyperhomocysteinaemia
IS: Ischemic stroke
ML: Machine learning
PCR: Polymerase chain rection
PPPM/3PM: Predictive preventive personalised medicine
ROS: Reactive oxygen species
SBI: Silent brain infarction
SCA: Sudden cardiac arrest
SCD: Sudden cardiac death
